# Horizontal Acquisition and Transcriptional Integration of Novel Genes in Mosquito-Associated *Spiroplasma*

**DOI:** 10.1093/gbe/evx244

**Published:** 2017-11-21

**Authors:** Wen-Sui Lo, Chih-Horng Kuo

**Affiliations:** Institute of Plant and Microbial Biology, Academia Sinica, Taipei, Taiwan; Molecular and Biological Agricultural Sciences Program, Taiwan International Graduate Program, National Chung Hsing University and Academia Sinica, Taipei, Taiwan; Graduate Institute of Biotechnology, National Chung Hsing University, Taichung, Taiwan; Biotechnology Center, National Chung Hsing University, Taichung, Taiwan

**Keywords:** Mollicutes, *Spiroplasma*, symbiont, horizontal gene transfer (HGT), gene gain, gene expression

## Abstract

Genetic differentiation among symbiotic bacteria is important in shaping biodiversity. The genus *Spiroplasma* contains species occupying diverse niches and is a model system for symbiont evolution. Previous studies have established that two mosquito-associated species have diverged extensively in their carbohydrate metabolism genes despite having a close phylogenetic relationship. Notably, although the commensal *Spiroplasma diminutum* lacks identifiable pathogenicity factors, the pathogenic *Spiroplasma taiwanense* was found to have acquired a virulence factor *glpO* and its associated genes through horizontal transfer. However, it is unclear if these acquired genes have been integrated into the regulatory network. In this study, we inferred the gene content evolution in these bacteria, as well as examined their transcriptomes in response to glucose availability. The results indicated that both species have many more gene acquisitions from the Mycoides-Entomoplasmataceae clade, which contains several important pathogens of ruminants, than previously thought. Moreover, several acquired genes have higher expression levels than the vertically inherited homologs, indicating possible functional replacement. Finally, the virulence factor and its functionally linked genes in *S. taiwanense* were up-regulated in response to glucose starvation, suggesting that these acquired genes are under expression regulation and the pathogenicity may be a stress response. In summary, although differential gene losses are a major process for symbiont divergence, gene gains are critical in counteracting genome degradation and driving diversification among facultative symbionts.

## Introduction

Symbiotic bacteria represent a considerable portion of extant biodiversity and can drive the diversification of their hosts (Moran 2006; Zilber-Rosenberg and Rosenberg 2008; McFall-Ngai et al. 2013). As such, understanding the evolutionary processes that affect the ecological divergence of symbionts is important. It is well established that associations with eukaryotic hosts can lead to reductive evolution in symbiont genomes (Moran and Plague 2004; Ochman and Davalos 2006; Moran et al. 2008; Moya et al. 2008; McCutcheon and Moran 2011; Moran and Bennett 2014; Lo, Huang, et al. 2016). During the process, gene losses are driven by mutational biases toward deletions (Mira et al. 2001; Kuo and Ochman 2009a) and accelerated mutation accumulation resulting from elevated genetic drift (Hershberg et al. 2007; Kuo et al. 2009; Novichkov et al. 2009). The differential gene losses, as well as acquisition of novel genes through horizontal gene transfer (HGT), all contribute to the genetic diversification and can potentially promote ecological divergence (Pál et al. 2005; Sirand-Pugnet et al. 2007; Kuo and Ochman 2009b; Lo, Huang, et al. 2016).

Among the symbiotic bacteria that have been characterized, the genus *Spiroplasma* represents an interesting study system because it includes species that are harmless commensals, mutualists, reproductive parasites, and pathogens associated with a variety of arthropod hosts (Gasparich et al. 2004; Anbutsu and Fukatsu 2011; Lo, Huang, et al. 2016). The phenotypic variation provides a good opportunity to investigate the genetic factors contributing to ecological divergence. Two mosquito-associated species, *Spiroplasma diminutum* (Williamson et al. 1996) and *Spiroplasma taiwanense* (Abalain-Colloc et al. 1988), are particularly relevant. These two species overlap in their geographic distribution (i.e., northern Taiwan) and natural host range (i.e., *Culex* mosquitoes). Artificial infection experiments have been conducted to test the possibility of developing these bacteria into biocontrol agents of mosquitoes (Chastel and Humphery-Smith 1991). The results indicated that while *S. diminutum* could replicate inside its host, no apparent fitness effect was observed (Vorms-Le Morvan et al. 1991; Williamson et al. 1996). In comparison, *S. taiwanense* infection induced extensive tissue damage and decreased adult flight ability (Phillips and Humphery-Smith 1995), as well as reduced larval survival (Humphery-Smith, Grulet, Chastel 1991) and adult lifespan (Humphery-Smith, Grulet, Le Goff, et al. 1991; Vazeille-Falcoz et al. 1994).

To investigate the genetic factors contributing to the difference in pathogenicity, we performed comparative genomics studies of these two *Spiroplasma* species and their relatives (Lo, Ku, et al. 2013; Chang et al. 2014). These studies suggested that the pathogenicity of *S. taiwanense* may be attributed to the presence of *glpO*. This gene is linked to the production of reactive oxygen species and has been demonstrated as a major virulence factor in two *Mycoplasma* species that belong to different clades within this polyphyletic genus. The first study was in *Mycoplasma mycoides* (Pilo et al. 2005), which belongs to the Mycoides-Entomoplasmataceae clade and was derived from a *Spiroplasma* ancestor. The second study was in the more divergent *Mycoplasma pneumoniae* (Hames et al. 2009), which belongs to the Pneumoniae group that is outside of the Spiroplasma-Entomoplasmataceae-Mycoides clade. Intriguingly, although *glpO* was likely to be present in the ancestral *Spiroplasma* (and lost in *S. diminutum*), the *S. taiwanense glpO* was putatively acquired from a Mycoides-Entomoplasmataceae donor, rather than vertically inherited. Other than *glpO*, these two species also differ considerably in their carbohydrate metabolism genes. Although *S. diminutum* possesses the complete gene sets for utilization of trehalose, cellobiose, sucrose, and N-acetylmuramic acid (MurNAc), multiple genes in these pathways are pseudogenized or absent in *S. taiwanense*. The differentiation in carbohydrate metabolism pathways between closely related species was found in other *Spiroplasma* lineages and was hypothesized as an important factor in driving ecological divergence (Lo, Chen, et al. 2013; Lo, Gasparich, et al. 2015; Paredes et al. 2015).

In this work, we took advantage of the recent improvement in taxon sampling of available *Spiroplasma* genomes (Ku et al. 2014; Lo, Gasparich, et al. 2015; Lo, Lai, et al. 2015; Lo, Liu, et al. 2015; Paredes et al. 2015; Lo, Gasparich, et al. 2016) to conduct a fine scale analysis of gene content evolution. Furthermore, we utilized strand-specific RNA sequencing (RNA-Seq) to compare the gene expression of *S. diminutum* and *S. taiwanense* under different conditions of glucose availability. Glucose was chosen because the entire gene set for glucose utilization is conserved among all *Spiroplasma* species characterized, whereas genes involved in the metabolism of other sugars (e.g., trehalose, cellobiose, sucrose, etc.) have variable patterns of presence and absence, suggesting that glucose is a key nutrient (Lo, Huang, et al. 2016). Moreover, glucose is one of the circulating sugars in mosquito hemolymph (Hou et al. 2015). Finally, one empirical study has demonstrated that glucose is the preferred carbon source of *M. pneumoniae* (Halbedel et al. 2004), further supporting the importance of this sugar for Mollicutes. In addition to providing a global comparison of gene expression between these two mosquito symbionts, we are interested in investigating if those horizontally acquired genes have been integrated in the gene regulatory network. Taken together, we aim to better understand the divergence between closely related insect symbionts.

## Materials and Methods

### Comparative Genomics and Molecular Phylogenetics

The procedures for comparative genomics and molecular phylogenetics were based on those described in our previous studies (Ku et al. 2013; Lo, Chen, et al. 2013; Lo, Ku, et al. 2013; Chang et al. 2014; Lo, Gasparich, et al. 2015). The parameters of the bioinformatics tools were based on the default settings unless stated otherwise. Briefly, eight genomes from the Apis clade, two from the sister Mycoides-Entomoplasmataceae clade (i.e., *Mesoplasma florum* and *M. mycoides*), and two from the more divergent Citri-Chrysopicola-Mirum clade (i.e., *Spiroplasma chrysopicola* and *Spiroplasma eriocheiris*) were sampled for homologous gene identification by using OrthoMCL (Li et al. 2003) with a BLASTP (Camacho et al. 2009) e-value cutoff of 1 × e^−15^ and an inflation value of 1.5. The GenBank accession numbers of these genome sequences are provided in supplementary table S1, Supplementary Material online. The protein sequences of conserved single-copy genes were aligned using MUSCLE v3.8 (Edgar 2004), concatenated into a single alignment, and used to infer a maximum likelihood phylogeny using PhyML v3.0 (Guindon et al. 2003). To estimate the bootstrap support, we used the SEQBOOT program of PHYLIP v3.69 (Felsenstein 1989) to generate 1,000 replicates of the alignment, which were then processed by using PhyML. The consensus tree of these bootstrap results was inferred using the CONSENSE program of PHYLIP. The gene content of those eight Apis-clade genomes was examined in the context of the species phylogeny. The ancestral state of gene presence or absence was inferred based on the principle of parsimony by scoring the possible events of gene gains and losses manually.

To identify the putatively acquired genes, all *S. diminutum* and *S. taiwanense* genes were used to run BLASTP searches against the NCBI nonredundant database (Benson et al. 2015). Genes with at least two of the top-five hits from non-*Spiroplasma* species were considered as candidates. Five major genes of interest were subjected to molecular phylogenetic analysis with the homologs selected from the BLASTP hits in the NCBI nonredundant protein database (as of October 2017). The GenBank accession numbers are provided in supplementary table S1, Supplementary Material online. The procedures for alignment, phylogenetic inference, and bootstrap evaluation are the same as described earlier. To validate the results produced by the maximum likelihood approach, we performed Bayesian inference using MrBayes v3.1.2 (Ronquist and Huelsenbeck 2003). The amino acid substitution model was set to mixed with gamma-distributed rate variation across sites and a proportion of invariable sites. The number of rate categories for the gamma distribution was set to four. The Markov chain Monte Carlo analysis was set to run for 1,000,000 generations and sampled every 100 generations. The first 25% of the samples were discarded as the burn-in.

### Bacterial Strains, Growth Conditions, and RNA Extraction

The type strains of *S. diminutum* and *S. taiwanense* were purchased from the American Type Culture Collection. Single colonies were picked and cultured in modified R_2_ broth (Moulder et al. 2002), in which sucrose was replaced by 2% glucose, until the cell density reached ∼1 × 10^7^ CFU/ml. Subsequently, ∼1 × 10^6^ CFU cells were transferred to 25 ml of basal R_2_ medium with or without the glucose supplement (i.e., the “G+” and “G–” treatments, respectively). The cells were cultured at 30 °C with shaking at 125 rpm for 20 h, and harvested during the log phase by centrifugation at 25, 186 × *g* for 30 min at 4 °C. The pellet was washed twice using phosphate-buffered saline.

Three biological replicates were collected and each G+/G– treatment pair was derived from the same colony. One replicate was used for the RNA-Seq experiment and the other two were used for validation by quantitative reverse transcription polymerase chain reaction (qRT-PCR). The MasterPure RNA Purification Kit (Cat. No. MCR85102; Epicentre, USA) was used for RNA extraction. The sample quality was checked using Qubit fluorometer (Invitrogen, USA) and Agilent BioAnalyzer (Agilent, USA).

### Transcriptome Sequencing and Analysis

The strand-specific RNA-Seq library preparation and sequencing were processed by the core facilities of our institution (Academia Sinica, Taiwan; see Acknowledgments). For each sample, 4 µg of the purified total RNA was used as the starting material. The ribosomal RNA was depleted using the Ribo-Zero rRNA Removal Kit for Bacteria (Cat. No. MRZMB126; Illumina, USA) following the manufacturer’s instructions. Subsequently, the TruSeq Stranded mRNA Library Prep Kit (Cat. No. RS-122-2101; Illumina, USA) was used for sequencing library preparation as described later. The mRNA purification step, which uses the oligo-dT beads to capture polyA tails of eukaryotic mRNA, was skipped because this step is not necessary for bacterial mRNA samples. The rRNA-depleted RNA was fragmented and the first-strand cDNA was synthesized using SuperScript III reverse transcriptase (Cat. No. 18080-093; Invitrogen, USA) with dNTP and random primers according to the manufacturer’s instructions. The second-strand cDNA was generated using a dUTP mix. The double-stranded cDNA was subject to the addition of a single “A” base to the 3′-end, followed by ligation of the barcoded TruSeq adapter. The products were purified and amplified with 10 cycles of PCR to generate the final double-stranded cDNA library. For quality check, we used the KAPA Library Quantification Kit (Cat. No. KK4824; Kapa Biosystems, USA) and the High Sensitivity DNA Analysis Kit (Cat. No. 5067-4626; Agilent, USA). Finally, the four libraries were pooled in equal ratio and sequenced in one 1 × 151 bp run on MiSeq (Illumina, USA).

The reads were trimmed at the first position from the 5′-end that has a quality score of <20; reads that are <20 bp were discarded. The resulting reads were mapped to each genome using BWA v0.7.12 (Li and Durbin 2009). The mapping results were processed by using SAMtools v1.2 (Li et al. 2009) and BEDTools v2.17.0 (Quinlan and Hall 2010) to calculate the read counts. NOISeq (Tarazona et al. 2011) was used to normalize the read counts and to infer the probability of differential expression. We chose the Reads Per Kilobase per Million (RPKM) mapped reads method for normalization and enabled the length correction option. Genes with at least 2-fold differences in RPKM values and a probability of at least 0.95 were defined as differentially expressed. To test the effect of normalization method, we also used the Trimmed Mean of M and the Transcripts Per Kilobase Million mapped reads methods. Because both alternatives produced normalized counts with *R*^2^ > 0.99 when compared with the RPKM results, we report only the RPKM values.

To find antisense RNAs (asRNAs), we identified the genes with deviated antisense expression (i.e., data points with RPKM values that are larger than the upper quartile or smaller than the lower quartile by more than 1.5 interquartile range). In addition to whole genes, regions that are at least 50 bp in length and have antisense expression were also considered. These candidates were manually examined using Artemis (Carver et al. 2012) to remove the false-positives caused by overlapping genes on the opposite strand.

To identify noncoding RNAs (ncRNAs), we extracted the Illumina reads mapped to the intergenic regions and assembled those reads using VELVET v1.2.07 (Zerbino and Birney 2008). Due to the high coverage of our RNA-Seq data sets (i.e., ∼1 Gb reads for ∼1 Mb genome), we removed candidates with a low coverage to reduce noise and to find ncRNAs with relatively high expression levels. Additionally, the kmer length and the expected coverage were optimized for each data set based on an iterative process. For *S. diminutum*, the VELVETH kmer length was set to 39 and the additional parameter settings for VELVETG were “-read_trkg yes -exp_cov 1000 -cov_cutoff 500 -min_contig_lgth 100.” For *S. taiwanense*, the VELVETH kmer length was set to 47 and the additional parameter settings for VELVETG were “-read_trkg yes -exp_cov 4000 -cov_cutoff 400 -min_contig_lgth 100.” The Rfam database (Griffiths-Jones et al. 2003), ARAGORN (Laslett and Canback 2004), and Bcheck (Yusuf et al. 2010) were used to annotate the resulting contigs.

### Validation of the RNA-Seq Results by qRT-PCR

For each sample, 5 µg of total RNA was reverse transcribed using random hexamers (Invitrogen, USA) and the Superscript III First-Strand Synthesis System (Invitrogen, USA). The targets were amplified using the Power SYBR Green PCR Master Mix (Applied Biosystems, USA) and quantified using the Applied Biosystems 7900 HT Real-Time PCR System (Applied Biosystems, USA). Three technical replicates were used for each sample and the C_T_ values were determined using SDS v2.3 (Applied Biosystems, USA). For normalization, we used *rpoD*, *tdk*, and *upp* as internal control. These three housekeeping genes exhibited relatively consistent expression in our RNA-Seq experiment (i.e., minimum/average/maximum RPKM fold-change = 0.79/0.96/1.16).

## Results and Discussion

### Gene Content Evolution in the Apis Clade

**Fig. 1.**
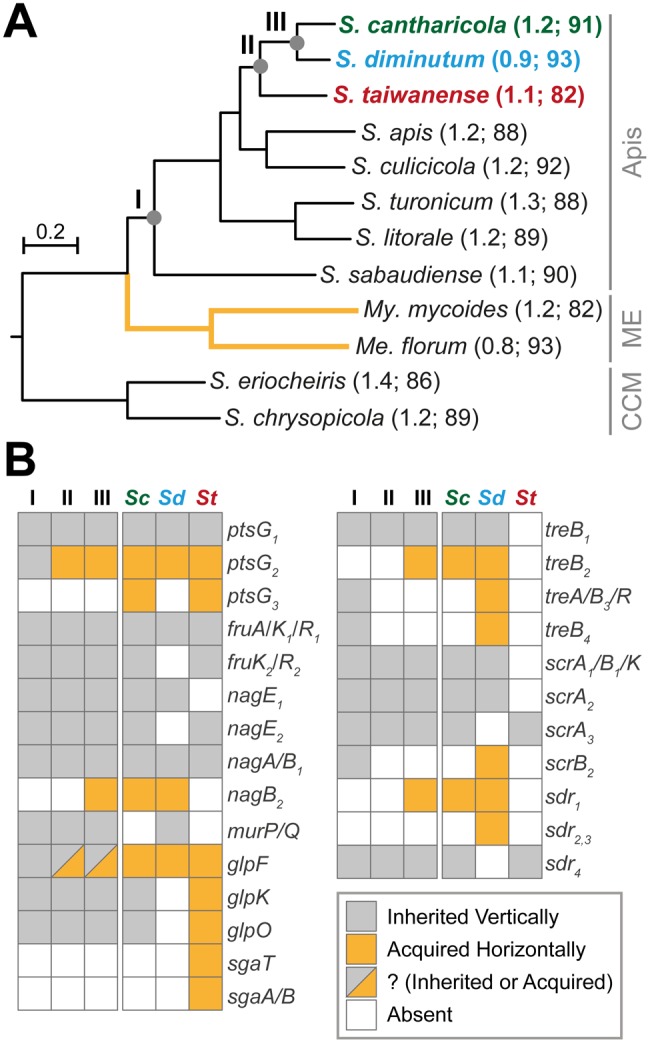
—Gene content evolution in the Apis clade. (*A*) Maximum likelihood species phylogeny based on 330 single-copy genes conserved in all 12 species. The concatenated protein alignment contains 120,314 aligned sites. All internal nodes received 100% bootstrap support based on 1,000 replicates. Three ancestral nodes of interest (i.e., I, II, and III) are labeled. The chromosome size (unit: Mb) and coding density (unit: %) of each genome are provided in parentheses following the species name. GenBank accession numbers of the sequences used are provided in supplementary table S1, Supplementary Material online. Clade label abbreviations: CCM, Citri-Chrysopicola-Mirum; ME, Mycoides-Entomoplasmataceae. (*B*) Patterns of gene presence and absence inferred for the three ancestral states and observed among the three extant species. Cells indicating gene presence are colored according to the inferred origin. Genes sharing the same gene name but were assigned to different homologous gene clusters are distinguished by numerical subscripts.

Based on a concatenated protein alignment of 330 conserved single-copy genes (with 120,314 aligned sites), we reconstructed a species phylogeny with 100% bootstrap support for all branches (fig. 1*A*). The topology is consistent with a previous genome-scale phylogeny that included more non-*Spiroplasma* lineages within the class Mollicutes (Bolaños et al. 2015). The ancestral gene content was inferred for three time points, including: (I) the most recent common ancestor (MRCA) of the Apis clade, (II) the MRCA of *S. cantharicola*–*S. diminutum*–*S. taiwanense*, and (III) the MRCA of *S. cantharicola*–*S. diminutum* (fig. 1*B*). The results indicated multiple gene gains and losses. In *S. diminutum*, the losses mostly involved redundant genes. For example, the losses of *fruK_2_*, *nagE_2_*, *scrA_3_*, and *sdr_4_* did not impact its substrate utilization capacity because other homologs have been maintained or acquired. The only notable exception is the loss of glycerol utilization genes (*glpK* and *glpO*), which likely explains its lack of pathogenicity (Lo, Ku, et al. 2013; Chang et al. 2014). In comparison, *S. taiwanense* has lost genes involved in the utilization of MurNAc (*murP* and *murQ*), trehalose (*treA* and *treB*), and sucrose (*scrB* and *scrK*). Because these losses are not complemented by other functional homologs, *S. taiwanense* has a narrow range of substrate utilization profile compared with other Apis clade species (Lo, Ku, et al. 2013; Chang et al. 2014).

In addition to these gene losses, *S. taiwanense* is notable in exhibiting signs of having experienced recent events of genome degradation. For example, *S. taiwanense* has a lower coding density (81.7% compared with ∼88–93%) and more annotated pseudogenes (65 compared with mostly <10) than other Apis clade species (fig. 1*A* and supplementary table S1, Supplementary Material online). This observation may be explained by an elevated level of genetic drift experienced by this species, which resulted in higher probabilities of mutation fixation. Consistent with this hypothesis, *S. taiwanense* was only isolated from *Culex tritaeniorhynchus* (Abalain-Colloc et al. 1988), whereas *S. diminutum* was isolated from C. *tritaeniorhynchus* and *Culex annulus* (Williamson et al. 1996). The narrower host range of *S. taiwanense* may correspond to a smaller effective population size and a higher level of genetic drift. Additionally, *S. taiwanense* reduces the mobility and survival of the infected mosquitoes (Humphery-Smith, Grulet, Chastel 1991; Humphery-Smith, Grulet, Le Goff, et al. 1991; Vazeille-Falcoz et al. 1994; Phillips and Humphery-Smith 1995), which could lower the transmission probability of this bacterium, further reducing its effective population size.

**Fig. 2.**
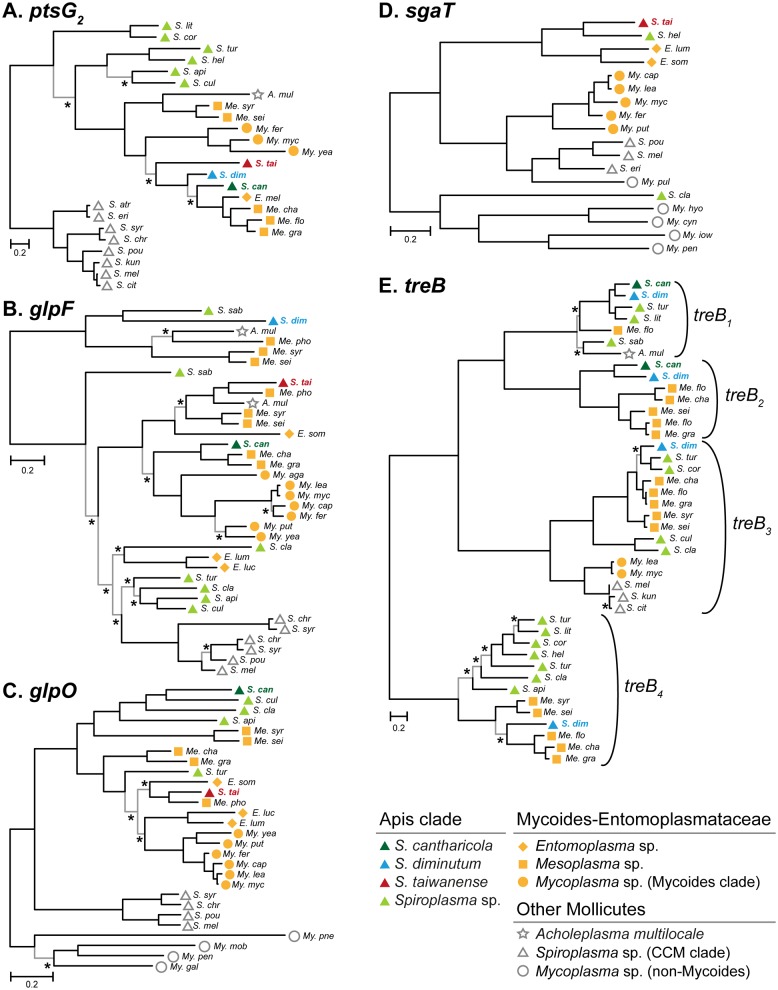
—Unrooted maximum likelihood phylogenies of putatively acquired genes. (*A*) *ptsG*_2_. (*B*) *glpF*. (*C*) *glpO*. (*D*) *sgaT*. (*E*) *treB*. The branches that received >75% bootstrap support and >95% Bayesian posterior probability support are colored in black; those fail either one of the criteria are colored in gray and indicated by asterisks. GenBank accession numbers of the sequences used are provided in supplementary table S1, Supplementary Material online.

Other than losses, gene gains through HGT were identified (fig. 1*B* and supplementary table S2, Supplementary Material online; 44 for *S. diminutum* and 40 for *S. taiwanense*). These acquired genes could complement gene losses, replace existing genes, increase gene dosages, or introduce novel functions. For example, while most of the Apis clade species have two copies of glucose transporter genes (*ptsG*), the second copy in *S. cantharicola*–*S. diminutum*–*S. taiwanense* appeared to be replaced by a homolog acquired from the Mycoides-Entomoplasmataceae clade (fig. 2*A*). Moreover, *S. cantharicola* and *S. taiwanense* both have a third copy of *ptsG* that lacks homolog in other Apis clade lineages (fig. 1*B*), which represents a copy number increase through HGT. The second example of notable HGT involves the glycerol utilization gene cluster (*glpF*/*K*/*O*). Our previous study suggested the presence of this gene cluster in the MRCA of the Apis clade (i.e., node “I” in fig. 1) and the homolog replacement in *S. taiwanense* by HGT (Chang et al. 2014). Intriguingly, as the taxon sampling of available Mollicutes genome sequences improves, this hypothesis became better supported and we also inferred that the *glpF* homologs in *S. cantharicola* and *S. diminutum* (fig. 2*B*) and the *glpO* homolog in *S. turonicum* (fig. 2*C*) were replaced through independent HGT events as well. Furthermore, the ascorbate transporter gene *sgaT* may have been acquired by different Apis clade lineages through at least two independent HGT events (fig. 2*D*). Finally, the phylogeny of *treB* homologs revealed a more complex evolutionary history (fig. 2*E*). Among the four copies of *treB* in *S. diminutum*, only *treB_1_* was inherited vertically. For the other copies, *treB_2_* might be acquired in the MRCA of *S. cantharicola* and *S. diminutum* (i.e., node “III” in fig. 1), *treB_3_* appeared to be acquired based on the sequence similarity to multiple *Mesoplasma* homologs (i.e., the *treB_3_* homologs in *S. culicicola* and *S. clarkii* are more likely to be inherited), and *treB_4_* was acquired from a *Mesoplasma* donor as well (while most of the other Apis clade lineages maintains a vertically inherited copy of *treB_4_*). Taken together, these results highlighted the extensive HGT events between two major clades of Mollicutes (i.e., Apis and Mycoides-Entomoplasmataceae). This pattern of dynamic gene content evolution has been reported in other facultative insect symbionts (Lo, Huang, et al. 2016) and is distinct from the lack of HGT observed among obligate endosymbionts (Ochman and Davalos 2006; Moran et al. 2008; McCutcheon and Moran 2011).

### Overview of the RNA-Seq Data Sets

Our RNA-Seq experiment generated ∼27 million raw reads (NCBI accessions SRR3466528–SRR3466531). After quality filtering, the overall mapping rate is ∼97.8%. The numbers of mapped reads range from 6.1 to 6.8 million per library, with ∼99% of these reads mapped to annotated features (table 1).

**Table 1:** Summary of the RNA-Seq Mapping Results

Genomic Features	*S. diminutum*		*S. taiwanense*	
	G+	G–	G+	G–
Protein-coding; sense	6,156,996 (93.2)	6,301,927 (92.6)	5,494,543 (90.0)	5,352,681 (85.1)
Protein-coding; antisense	19,068 (0.3)	20,452 (0.3)	24,391 (0.4)	51,600 (0.8)
Protein-coding; pseudogene	—	—	87,893 (1.4)	175,470 (2.8)
tmRNA	210,223 (3.2)	222,244 (3.3)	305,582 (5.0)	453,486 (7.2)
RNaseP (*rnpB*)	102,521 (1.6)	155,919 (2.3)	95,023 (1.6)	149,237 (2.4)
rRNA	3,278 (0.1)	5,308 (0.1)	10,967 (0.2)	31,913 (0.5)
tRNA	26,829 (0.4)	26,091 (0.4)	26,033 (0.4)	12,590 (0.2)
Intergenic	88,427 (1.3)	70,859 (1.0)	61,894 (1.0)	61,278 (1.0)

Note.—Numbers indicate the counts and percentages (in parentheses) of the RNA-Seq reads mapped to specific features in each genome under the two experimental conditions (i.e., G+ and G–).

**Fig. 3.**
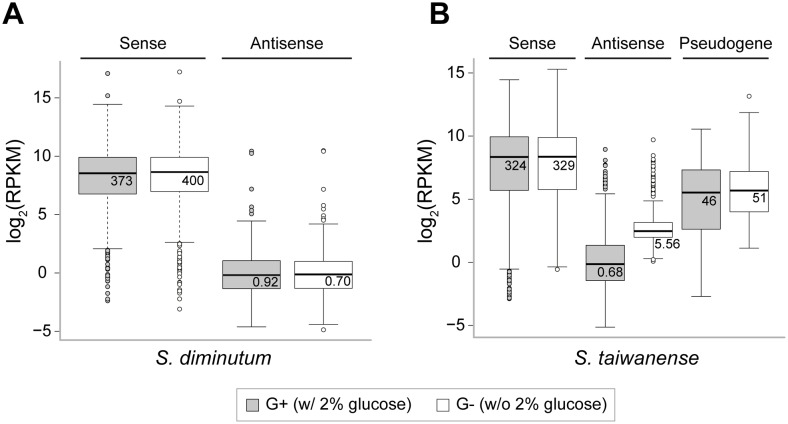
—Distribution of the RPKM values observed. (*A*) *Spiroplasma diminutum*. (*B*) *Spiroplasma taiwanense*. The median values prior to log-transformation are labeled. The inner fence values (i.e., ends of the whiskers) represent the lowest value within 1.5 interquartile range of the lower quartile and the highest value within 1.5 interquartile range of the upper quartile.

Only ∼0.05–0.50% of the reads were mapped to the rRNA genes, indicating that the rRNA removal was highly effective. Approximately 85–93% of the reads were mapped to the sense strand of protein-coding genes. In comparison, <1% were mapped to the antisense strand (table 1). The expression levels of sense and antisense strands differ by two orders of magnitude; the median RPKM values are 324–400 and 0.7–5.6 for sense and antisense strands, respectively (fig. 3 and supplementary table S3, Supplementary Material online). To investigate if any of the asRNAs may be involved in expression regulation, we compared the RPKM values of these asRNAs between the two growth conditions. In *S. diminutum*, none of the asRNA candidates reached our criteria for differential expression. In *S. taiwanense*, although a higher percentage of reads were mapped to the antisense strand in the G– condition (table 1), only nine candidates reached our criteria of significance. All these nine asRNAs correspond to hypothetical proteins located on the plasmid. Thus, the functional significance is unclear and it is likely that most of the asRNA expression we observed were resulted from inefficient transcription control (Nicolas et al. 2012; Raghavan et al. 2012; Lloréns-Rico et al. 2016).

**Fig. 4.**
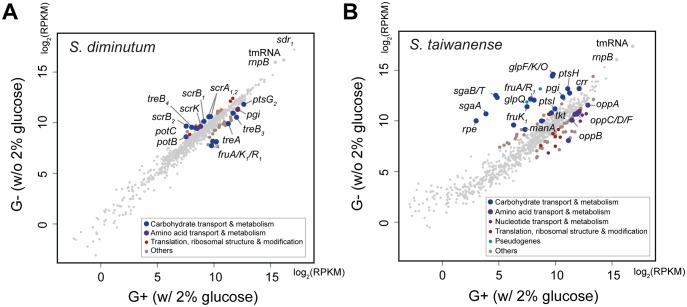
—Gene expression levels in response to glucose availability. (*A*) *Spiroplasma diminutum*. (*B*) *Spiroplasma taiwanense*. The expression levels are measured in RPKM mapped reads (log_2_-transformed). All protein-coding genes and two highly expressed noncoding genes (i.e., tmRNA and *rnpB*) are shown. The genes exhibiting significant differential expressions between the two growth conditions are color-coded based on their COG functional category assignments. The remaining genes are represented by gray dots.

For ncRNAs, we identified two loci exhibiting high expression levels in both species. The first locus is a transfer-messenger RNA (tmRNA), which is conserved among most bacteria (Hudson et al. 2014). In both species, the tmRNA locus is adjacent to the small protein B gene (*smpB*). The products of these two genes form the tmRNA–SmpB complex that functions as the primary rescue system for ribosome stalling (Keiler 2007; Himeno et al. 2014). The second ncRNA locus corresponds to a ribonuclease P (RNase P) gene (*rnpB*), which is an important component of RNA processing (Kazantsev and Pace 2006). These two ncRNAs had higher expression levels than all protein-coding genes (fig. 4). However, no significant differential expression was observed.

Finally, approximately 1% of the reads were mapped to intergenic regions. Because the RPKM values were low and no significant differential expression was found, it is likely that most of these reads were the results of inefficient transcription control. For these reasons, our RNA-Seq analysis below focused on the sense-strand of protein-coding genes.

### Expression Profiles of *S. diminutum*

**Fig. 5.**
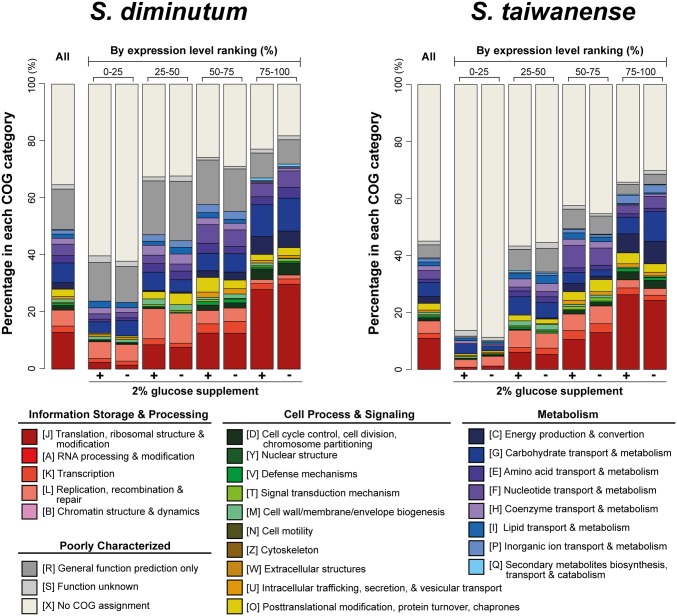
—Functional category distribution of protein-coding genes. The functional categories are assigned based on the COG database. In addition to the overview (“All”), the protein-coding genes in each genome are divided into four groups according to their percentile ranking of expression levels.

**Fig. 6.**
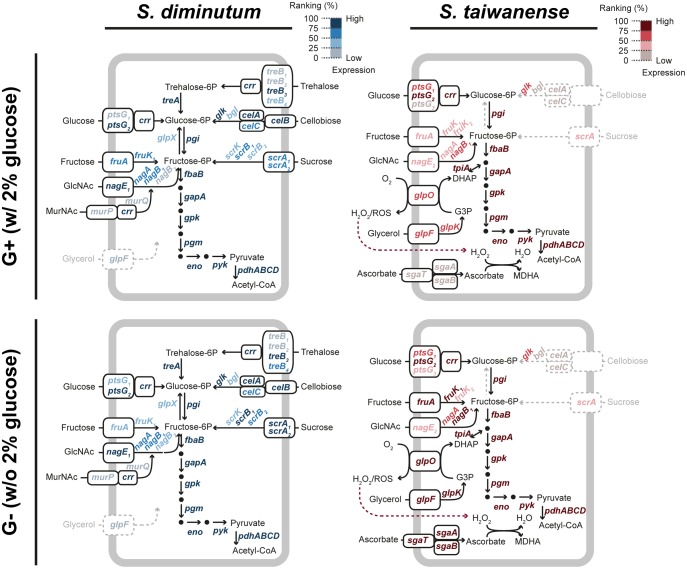
—Expression profiles of carbohydrate metabolism genes. The genes are color-coded based on the ranking of expression levels. Substrates and pathways that lack the complete sets of required genes are presented in gray.

In *S. diminutum*, ∼93% of the RNA-Seq reads were mapped to the protein-coding genes in both conditions (table 1). Approximately 95% of these genes exhibited similar expression levels (fig. 4*A*). In general, highly expressed genes were enriched with those involved in translation, energy production, and carbohydrate metabolism. In contrast, genes with lower expression levels were dominated by those with poorly characterized functions (fig. 5). The entire pathway for carbohydrate catabolism (*treA*, *pgi*, *fbaB*, *gapA*, *gpk*, *pgm*, *eno*, *pyk*, and *pdhABCD*) and several carbohydrate transporter genes (*ptsG_2_*, *crr*, *nagE_1_*, *treB_3_*, and *celA/B*) were among the highly expressed genes that ranked at 75th percentile or higher in both conditions (fig. 6). These findings further support the importance of carbohydrate metabolism in *Spiroplasma* (Carle et al. 2010; Lo, Chen, et al. 2013; Lo, Ku, et al. 2013; Chang et al. 2014; Lo, Gasparich, et al. 2015; Paredes et al. 2015). These highly expressed transporter genes corresponded well with the sugars that may be available from the insect hosts. For example, trehalose and glucose are the major sugars in insect hemolymph, cellobiose and sucrose could be acquired from the nectar in host diet, and N-acetylglucosamine (GlcNAc) could be derived from the chitinous peritrophic matrix in host midgut.

**Fig. 7.**
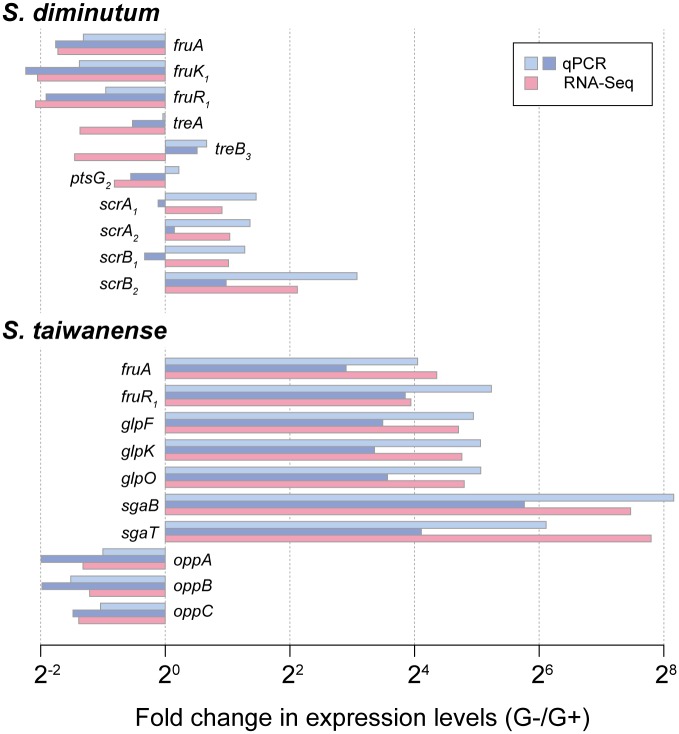
—Validation of differential expression in selected genes. Three biological replicates were collected for this study. The first set was used in the RNA-Seq experiment for the initial identification of differentially expressed genes, the remaining two were used for validation by qRT-PCR.

Comparing with the nutrient-rich G+ condition, 20 and 26 genes exhibited significantly higher and lower expression level in the G– condition, respectively. Among these 46 differentially expressed genes, 15 are related to carbohydrate catabolism (fig. 4*A*). Several genes for glucose (*ptsG_2_*), trehalose (*treA* and *treB_3_*) and fructose (*fruA*/*K_1_*/*R_1_*) metabolism exhibited higher expression levels under in the G+ condition, whereas the entire set of sucrose utilization genes (*scrA_1_*, *scrA_2_*, *scrB_1_*, *scrB_2_*, and *scrK*) and one of the trehalose transporter genes (*treB_4_*) exhibited higher expression in the G– condition. To verify these results from RNA-Seq, we checked the mRNA abundance of 10 selected genes in two other biological replicates by qRT-PCR. The patterns observed for *fruA*/*K_1_*/*R_1_* and *scrB_2_* were strongly supported (fig. 7), whereas the remaining ones received weaker support (*treA*, *scrA_1_*, *scrA_2_*, and *scrB_1_*) or exhibited inconsistent results (*treB_3_*). This inconsistency may be caused by the different normalization methods used in the two approaches. For RNA-Seq, the expression levels were normalized by the total read counts across the entire genome. In comparison, the qRT-PCR protocol utilized only three reference genes for normalization, which may suffer from higher noise levels and unable to detect subtler changes in the expression levels of target genes. In support of this inference, the genes that received strong support in qRT-PCR validation all have an expression level difference of approximately 4-fold or higher, whereas the remaining ones all have an expression level difference of less than 2.9-fold. Furthermore, for the differentially expressed genes in *S. taiwanense*, all examined candidates exhibited large differences in expression levels and received strong support from qRT-PCR validation (fig. 7).

Other than carbohydrates, lipids are also important for *Spiroplasma* to acquire from their hosts. For example, proliferation of the *Drosophila*-associated *Spiroplasma poulsonii* is limited by the lipid availability of the host hemolymph (Herren et al. 2014). In *S. diminutum*, the gene with the highest expression level in both conditions (fig. 4*A*) is a short-chain dehydrogenase/reductase (*sdr_1_*; locus tag: SDIMI_v3c04970) that contains conserved domains for NADP binding site and steroid binding site. This finding suggests that *S. diminutum* may acquire energy through dehydrogenizing steroids, which are essential components of artificial culture medium (Williamson et al. 1996). Intriguingly, this gene has a homolog in *S. cantharicola* but not other Apis clade species, indicating that it may have been acquired through HGT in the MRCA of *S. cantharicola* and *S. diminutum* (i.e., time point III in fig. 1*A*). Although there are other genes annotated as short-chain dehydrogenase/reductase in *S. diminutum* and other Apis clade species, those genes lack the steroid binding site and exhibited much lower levels of expression (i.e., RPKM = 63–460, compared with ∼140,000 for SDIMI_v3c04970; supplementary table S3, Supplementary Material online), suggesting that they have different functional roles.

### Expression Profile of *S. taiwanense*

In *S. taiwanense*, 90.0% and 85.1% of the RNA-Seq reads were mapped to the protein-coding regions in the G+ and the G– condition, respectively (table 1). The difference is mainly due to a higher proportion of the reads that were mapped to the regions encode for tmRNA, pseudogenes, and RNaseP in the G– condition. As stated above, the differences in expression levels for tmRNA and RNaseP were not significant. Almost all of the 65 annotated pseudogenes had low expression levels and were not differentially expressed (fig. 3*B* and supplementary table S3, Supplementary Material online). Two notable exceptions are both related to glycerol metabolism, including *glpQ* and *glpU*. Both genes exhibited similar patterns of ∼20-fold higher expression levels in the G– condition as observed in the upstream *glpF*/*K*/*O* (supplementary table S3, Supplementary Material online). However, *glpQ* and *glpU* have been disrupted by frameshift mutations and have RPKM values that are <40% of *glpF*/*K*/*O*, suggesting that these two pseudogenes are no longer functional (Kuo and Ochman 2010). For the intact protein-coding genes, the patterns for overall functional category distribution based on the expression levels were similar to those observed in *S. diminutum* (fig. 5). For example, genes involved in translation, energy production, and carbohydrate metabolism are enriched among the highly expressed genes, whereas >80% of the genes with low expression levels are those without any COG functional category assignments. Based on the observations that >100 of these putative genes with low expression levels are <600 bp in length and lack any functional annotation, many of these sequences may be fragments of pseudogenes that are yet to be identified. However, in the absence of identifiable full-length homologs, it is difficult to further improve the annotation of pseudogenes. Nonetheless, these results suggest that the gene number and coding density are likely to be overestimated.

A total of 92 genes reached our threshold of differential expression. All 10 genes selected for validation by qRT-PCR produced consistent results among the three biological replicates (fig. 7), possibly because the expression level differences between the two conditions were much higher than those observed in *S. diminutum*. Interestingly, despite having twice as many differentially expressed genes compared with *S. diminutum*, the major glucose transporter gene *ptsG_2_* in *S. taiwanense* was not differentially expressed (STAIW_v1c06580; RPKM values = 12,144 and 10,962 in the G+ and G– conditions, respectively). The 52 genes that showed significantly lower expression levels in the G– condition included five for translation, six for amino acid metabolism, and five for nucleotide metabolism, suggesting that the cells may have lower rates of protein synthesis and DNA replication under glucose starvation. Consistent with this observation, the total RNA yield of the G– sample was only ∼12% of the G+ sample in *S. taiwanense*, indicating a severe reduction in growth. In comparison, the total RNA yield of the G– sample was ∼41% of the G +  sample in *S. diminutum*, suggesting that the more flexible carbohydrate metabolism of this species allows it to be less affected by glucose starvation.

In *S. taiwanense*, 40 genes have significantly higher expression levels in the G– condition, including 19 associated with carbohydrate metabolism such as those for fructose (*fruA*, *fruK_1_*, *fruR_1_*, *manA*, *rpe*, and *tkt*), glycerol (*glpF*/*K*/*O*), and ascorbate (*sgaA/B/T*) (figs. 4*B* and 6). The differential expression of these fructose/glycerol utilization genes is similar to the effect of carbon catabolite repression commonly found among bacteria (Deutscher et al. 2006; Görke and Stülke 2008; Fujita 2009), in which the genes for less preferred carbon sources are repressed in the presence of the preferred one. However, the global transcriptional regulator of carbon catabolite repression *ccpA* is absent in *Spiroplasma* and the related *Mycoplasma*. Instead, based on previous studies of *M. pneumonia*, the regulation of carbohydrate metabolism is controlled by *hprK*, which in turn is modulated by the concentration of ATP, Pi, and glycolytic intermediates (Steinhauer et al. 2002; Halbedel et al. 2004).

The differential expression of glycerol metabolism genes in *S. taiwanense* is worth noting due to the link to Mollicutes pathogenicity (Pilo et al. 2005; Hames et al. 2009). Intriguingly, the virulence factor *glpO* was found to be constitutively expressed in *M. pneumonia* and not modulated by the presence of glycerol or glucose (Hames et al. 2009), whereas here we demonstrated that glucose starvation could increase *glpF/K/O* expression by ∼27-fold in *S. taiwanense*. This finding may be relevant to its gene regulation in response to changes in host physiology. In female *Aedes aegypti* mosquitoes, having a blood meal could induce dramatic changes in the carbohydrate metabolism, reducing the trehalose, glucose, and fructose circulating in hemolymph by half at 6 h postblood meal (Hou et al. 2015). In comparison, the relative abundance of triacylglycerols remains at ∼80% at the same time point. Thus, it is possible that the gene expression changes observed in *S. taiwanense* represents a stress response toward sugar starvation by switching to glycerol as an alternative carbon/energy source, and the resulting pathogenicity toward hosts is a by-product of this strategy. Moreover, the ascorbate transporter genes *sgaA*/*B*/*T* were found to have >100-fold higher expression levels in the G– condition, which may help *S. taiwanense* to counter the oxidative stress induced by GlpO activity (figs. 4*B* and 6). In this context, it is interesting to note that the *sgaA/B/T* genes in *S. taiwanense* all appeared to have been acquired horizontally as well (figs. 1*B* and 2*D*). Because the *sgaA/B/T* cluster is located ∼50 kb upstream of the *glpF/K/O* cluster, it is likely that these two gene clusters were acquired through independent HGT events.

### Implications on the Genome Evolution of Facultative Symbionts

Driven in part by the rapid advancement in genomics, our understanding of symbiont evolution has improved dramatically over the past decade. One major topic in this field was the comparison with free-living bacteria. Initially, the general pattern emerged was that symbiont genome evolution is dominated by extensive gene losses, whereas gene gains through horizontal transfer are rare to none once the bacterium adopted a symbiotic lifestyle and has a genome that is <1.5 Mb in size (Ochman and Davalos 2006). Later on, a distinction was made between facultative symbionts with small yet dynamic genomes (e.g., *Spiroplasma* and *Wolbachia*, with the chromosomes being ∼1 Mb in size and containing mobile genetic elements) and obligate endosymbionts with tiny and stable genomes (e.g., insect bacteriome-restricted mutualists with the chromosomes being <0.5 Mb in size) (Moran et al. 2008). Among the facultative symbionts, although the incidences of HGT are rare compared with free-living bacteria, some of the acquired genes could lead to switches between ecological niches and have far-reaching implications on adaptation (Lo, Huang, et al. 2016). In this study, we found that as the taxon sampling of available genome sequences improves, our ability of detecting HGT among closely related lineages improves as well. For example, although the *S. taiwanense glpF/K/O* have been inferred as acquired, the *S. diminutum glpF* was assumed to be vertically inherited (Chang et al. 2014). With more genome sequences become available, it appears that the *S. diminutum glpF* may have been independently acquired from another Mycoides-Entomoplasmataceae clade donor (fig. 2*B*). Because lineages from these two clades share the same alternative genetic code, similar nucleotide composition biases, and similar ecological niches (e.g., insect gut and plant surface), it is plausible that more cases of HGT are yet to be discovered. Moreover, these factors that elevated probability of HGT could also contribute to the successful integration of acquired genes.

Our observation that most *Spiroplasma* HGT events involved nutrient metabolism genes is consistent with the complexity hypothesis (Jain et al. 1999; Cohen et al. 2011). This hypothesis asserts that genes with higher levels of connectivity in the protein–protein interaction networks are less likely to experience HGT. In our inference, the acquired genes all correspond to components that are peripheral in the metabolic network (figs. 1*B* and 6). The differential gains and losses of these peripheral genes, shaped by stochastic events and selection, eventually resulted in the different patchworks of gene content found among closely related lineages and their divergence in ecological niches.

Previous studies have shown that genes with high expression levels were less likely to be involved in HGT (Sorek et al. 2007; Park and Zhang 2012). However, in the case of these two *Spiroplasma* species, acquired genes are among some of the most highly expressed ones at RNA level. For example, an acquired short-chain dehydrogenase/reductase in *S. diminutum* has an expression level that far exceeds all other protein-coding genes in both conditions tested (fig. 4*A*). Moreover, in the cases of glucose transporters, the horizontally acquired *ptsG_2_* has an expression level that is >30-fold higher than the vertically inherited *ptsG_1_* in both species (fig. 6). This observation suggests that the selective pressure for maintaining *ptsG_2_* could be higher, which may eventually lead to homolog replacement as the case of *glpF*, in which only an acquired copy remains while the inherited one was lost. Finally, the *glpF/K/O* and *sgaA/B/T* gene clusters in *S. taiwanense* are particularly worth noting. The dramatic changes of expression levels in response to glucose availability (fig. 4*B*) indicate that these acquired genes are under regulatory control in the new genome.

## Conclusions

Taken together, although HGT is expected to be limited by ecological and functional barriers (Popa and Dagan 2011; Baltrus, 2013) and was thought to be rare among symbionts, case studies like this and others (Lo, Huang, et al. 2016) provide mounting evidence that HGT is an important evolutionary process for the evolution of facultative symbionts. The acquired genes may counter gene losses, contribute to gene family expansion, or even introduce novel functions. It is possible that the successful integration of few acquired genes could lead to a shift in the type of associations (e.g., commensalism to parasitism or parasitism to mutualism) (Lo, Huang, et al. 2016). In summary, this study demonstrated that continuing improvement in the taxon sampling of complete genome sequences available increases the power for detecting HGT among closely related species, which may have occurred more frequently than previously thought. Additionally, the comparative transcriptomics approach provides a high-throughput tool for examining the integration of acquired genes into the gene regulatory network, which facilitates the functional inference and improves our understanding of symbiont genome evolution. For future work, comparative metatranscriptomic experiments that examine the gene regulation of host and symbiont together could further improve our understanding of the whole system.

## Supplementary Material

Supplementary data are available at *Genome Biology and Evolution* online.

## Supplementary Material

Supplementary Tables
